# Clinical Alarms in intensive care: implications of alarm fatigue for the safety of patients^1^

**DOI:** 10.1590/0104-1169.3488.2513

**Published:** 2014

**Authors:** Adriana Carla Bridi, Thiago Quinellato Louro, Roberto Carlos Lyra da Silva

**Affiliations:** 2 Doctoral student, Universidade Federal do Estado do Rio de Janeiro, Rio de Janeiro, RJ, Brazil; 3 Doctoral student, Universidade Federal do Estado do Rio de Janeiro, Rio de Janeiro, RJ, Brazil. Assistant Professor, Universidade Federal Fluminense, Rio das Ostras, RJ, Brazil; 4 PhD, Adjunct Professor, Escola de Enfermagem Alfredo Pinto, Universidade Federal do Estado do Rio de Janeiro, Rio de Janeiro, RJ, Brazil

**Keywords:** Nursing, Intensive Care, Monitoring, Clinical Alarms, Iatrogenic Disease, Patient Safety

## Abstract

**OBJECTIVES:**

to identify the number of electro-medical pieces of equipment in a coronary care unit, characterize their types, and analyze implications for the safety of patients from the perspective of alarm fatigue.

**METHOD:**

this quantitative, observational, descriptive, non-participatory study was conducted in a coronary care unit of a cardiology hospital with 170 beds.

**RESULTS:**

a total of 426 alarms were recorded in 40 hours of observation: 227 were triggered by multi-parametric monitors and 199 were triggered by other equipment (infusion pumps, dialysis pumps, mechanical ventilators, and intra-aortic balloons); that is an average of 10.6 alarms per hour.

**CONCLUSION:**

the results reinforce the importance of properly configuring physiological variables, the volume and parameters of alarms of multi-parametric monitors within the routine of intensive care units. The alarms of equipment intended to protect patients have increased noise within the unit, the level of distraction and interruptions in the workflow, leading to a false sense of security.

## Introduction

Are alarms really a good fit for intensive care? Even though it seems paradoxical, this question has gained meaning due to the results of studies, especially international studies, which have recently indicated that the presence of a high number of alarms pose a potential risk to the integrity and safety of patients in intensive care units.

This is not only due to organic disorders caused by high levels of noise but also because it leads professionals to become desensitized, decreasing alertness and confidence in the urgency of these alarms resulting in what is called alarm fatigue. 

This phenomenon occurs when a large number of alarms mask other clinically significant ones so that some important alarms are disabled, silenced or ignored by the staff, compromising the safety of patients with severe conditions under intensive care. A lack of response to relevant alarms may result in severe consequences for the clinical conditions of patients^(^ 1 ^)^.

Deactivation of alarms, not programing or not properly configuring alarms in accordance with a patient's clinical condition and also setting them at a low volume are objects of research^(^ 2 ^)^. Professionals describe alarms as being "noisy, blatant, a nuisance" requiring the need to interrupt the care being provided to patients in order to attend to alarms^(^ 3 ^)^.

There is a high incidence of false alarms in intensive therapy units due to monitoring systems characterized by high sensitivity and low specificity. There is an excessive number of such alarms with low clinical relevance^(^ 1 ^)^.

A lack of standardization of alarm sounds, as to what an appropriate urgent alarm is, and inadequate visual and audio elements in a monitor's alarms, all have been objects of investigation in the nursing field^(^ 4 ^)^.

In regard to the equipment, researchers note that the complex programing, configuration and operation of alarm systems pose difficulties for staff^(^ 4 ^)^. Failures in equipment that leads to adverse events in intensive care units are described in the literature as important factors impacting the safety of patients^(^ 5 ^)^. 

In terms of human resources, studies show that professionals lack training on how to handle equipment correctly, that there is a deficit of human resources in units, a lack of adherence on the part of the staff in programming and configuring alarms and a lack of confidence in the urgency of alarms^(^ 4 ^)^.

Also, the physical disposition of units, which is inadequate to attend properly to alarms, a lack of maintenance of equipment and the involvement of the health staff and clinical engineering, have been investigated^(^ 4 ^)^.

Data from 2005 to 2008 show that the Food and Drug Administration (FDA) and the Manufacturer and User Facility Device Experience (MAUDE) received 566 reports of patient deaths related to monitoring alarms in hospitals in the United States of America (USA). There were, between March and June 2010, more than 73 deaths related to alarms, 33 of which were multi-parametric monitors^(^ 6 ^)^.

The Emergency Care Research Institute (ECRI), an organization specializing in patient safety and the use of electro-medical equipment, listed the 10 dangers of technology in the health field and alarms was in the number one danger in 2012 and 2013 due to the high number of adverse events among inpatients of hospitals in the USA, including death, cardiorespiratory arrest, and cardiac arrhythmias^(^ 7 ^)^_._

Based on data involving adverse events caused by alarms, the Joint Commission proposed that, for 2014, the management of clinical alarms should be pursued in order to improve the safety of these systems^(^ 8 ^)^. It is worth noting that discussions of this subject in Brazil are still incipient and mainly developed by the research group to which the authors of this study belong.

Considering the importance of this topic, we verified the need to gain results able to ground strategies to improve the monitoring systems used in the follow-up of critical patients under intensive care and to minimize alarm fatigue, so that monitoring is more objective and safe.

The study's objectives included: to identify the number of alarms from electro-medical equipment in a coronary care unit; to characterize the types of alarms; and to analyze implications for the safety of patients from the perspective of alarm fatigue.

## Method

This quantitative observational study was conducted in a coronary care unit (CCU) of a public university cardiology hospital with 170 beds, located in a city in the Southern region of Brazil.

We observed the production of data in five beds (beds 1 to 5) of the 12 beds available in the unit. This convenience sample enabled the observation and reliable counting of all the alarms that went off during the observation period. These beds are reserved for the most critical and unstable inpatients in the unit who require monitoring of physiological variables given the complexity of their conditions and the use of hemodynamic, ventilator and mechanical support. We took into account hemodynamic support (consisting of drips with vasoactive, antiarrhythmic, anti-hypertensive and inotropic medications); mechanical support (use of intra-aortic balloons); and ventilator support (use of invasive mechanical ventilation). A total of 49 patients in the day shift (DS) and 39 patients in the night shift (NS), monitored and using support, were observed during the period of data collection, totaling 88 patients.

The beds selected for the sample are equipped with multi-parametric monitors - AGILENT^(r)^ V26C/anesthesia - with numerically adjusted volume, from 0 to 255 dB, a visual signal (a light) of the physiological variable being monitored, idiomatic Portuguese, and a pause of 3 minutes between alarms. The unit does not have a central monitor. The mechanical ventilators are SERVO S^(r)^ ventilators, the infusion pumps are BBRAUN INFUSOMAT COMPACT^(r)^ pumps, and the intra-aortic balloons are Datscope 97Es^(r)^.

Observation totaled 40 non-continuous hours that took place on different days and at different times between March and June, 2012: 20 hours of observation during the DS and 20 hours during the NS, between 7am to 6pm and between 7pm and 12am, respectively. This strategy was adopted to produce a variability of situations and routines in both shifts, trying to portray the shifts accurately and avoid biases.

Data were collected through the completion of a form intended to collect observation data, where information concerning the patients under observation and their monitoring were recorded: clinical diagnosis, therapeutic support, physiological variables monitored (heart rate, electrocardiographic tracing-arrhythmias/ECG, non-invasive blood pressure/NIBP, mean invasive blood pressure/IMBP, respiratory, oxygen saturation/SpO2 and pulse), what alarms were enabled and their respective signal volumes. 

We used non-participatory observation (except in more critical intercurrences that had the potential to harm the patient), so that when an alarm went off we recorded the equipment from which the alarm originated: mechanical ventilators, infusion pumps, dialysis equipment, intra-aortic balloon, or multi-parametric monitors. The physiological variables that generated the alarms were also recorded. 

Data collected during the observation period and also concerning the patients were organized into a spreadsheet in Microsoft(r) Office Excel 2007 and later processed and analyzed using R version 2.15.1. Descriptive analysis was used for the study variables presenting mean, median, simple and absolute frequencies, and dispersion (interquartile range/ IQR)

This study met the guidelines set out by Resolution MS 196/96 and was approved by the hospital's Institutional Review Board (CEP/INC nº 0351/11-10-2011).

## Results

Hemodynamic support was utilized by 24 (32.08%) patients in the DS (n=49) and by 15 (12.40%) patients in the NS (n=39). Ventilator support was used by 37 (75.51%) patients in the DS and by 24 (61.54%) in the NS, indicating the complexity of the conditions of the patients observed in these periods.

The total number of alarms that went off from the multi-parametric monitors in the 40 hours of observation (20h in DS and 20h in the NS) was 227 (average of 5.7 alarms/hour), while 106 (average of 5.3 alarms/hour) alarms went off in the DS and 121 (average of 6.0 alarms/hour) in the NS. Note the high average number of alarms going off per hour in the service considering there are alarms from other equipment, together with environmental noise, and noise generated by the professionals themselves, which causes the environment to be stressful, heightening occupational risks and hindering patients' rest. Alarms in this environment are relevant but can be underestimated by the staff if muffled by other, less relevant, ones.

Other alarms were also observed, such as alarms from infusion pumps, dialysis, mechanical ventilators and intra-aortic balloons. A total of 199 alarms went off in a total of 40 hours of observation (an average of 4.9 alarms per hour). The following frequency was observed: 124 alarms in the DS (average of 6.2 alarms/hour) and 75 in the NS (average of 3.7 alarms/hour), which shows the high number of alarms present in the services. 

Therefore, a total of 426 alarms were recorded: 227 triggered by multi-parametric monitors and 199 triggered by other equipment (infusion pumps, hemodialysis, mechanic ventilators and intra-aortic balloons) in 40 hours of observation, an average of 10.6 alarms per hour, i.e., 11.5 and 9.8 hours in the day and night shifts, respectively. If not attended to, the alarms accumulate in the environment. The alarms last an average of 3 minutes and, if not attended to, they go off again so that we have 10 alarms in the first hour and, if these are not attended to, there will be 20 alarms in the second hour.

Table 1 shows the physiological variables monitored. ECG-arrhythmia and heart-rate monitoring were active for 100% of the patients observed in both the DS and NS, while nine (7.44%) of the 39 patients observed had their respiratory status monitored. Respiratory monitoring would detect any alteration in critical patients with a predisposition to unstable breathing conditions that require support.

**Table 1 -:** Profile of physiological variables monitored in the observed patients. Rio de Janeiro, RJ, Brazil, 2012

Physiological variables	Day Shifts (DS) (n = 49)	Night Shift (NS) (n = 39)
ECG – Arrhythmia monitoring*	49 (100%)	39 (100%)
Heart rate monitoring (%)	49 (100%)	39 (100%)
IABP^‡^ monitoring (%)	23 (46.94%)	10 (25.64%)
NIABP^§^ monitoring (%)	26 (53.06%)	29 (74.36%)
Pulse monitoring (%)	46 (93.88%)	38 (97.44%)
Respiratory monitoring (%)	30 (28.30%)	9 (7.44%)
SpO_2_^||^ (%)	46 (93.88%)	38 (97.44%)

n=Total of monitored patients under observation in the DS (n=49) and NS (n=39);

* ECG - Arrhythmia: electrocardiographic tracing;

‡ IABP: Invasive Arterial Blood Pressure;

§ NIABP: Non-Invasive Arterial Blood Pressure;

|| SpO2: Oxygen saturation.

Table 2 shows the profile of alarms that were enabled among the observed patients. A low absolute number and low percentage was found of equipment monitoring arrhythmia, pulse, respiratory and oxygen saturation, the alarms of which were enabled in both periods. This information reveals that, even though arrhythmia and heart rate were being monitored among all the patients under observation, not all the alarms were enabled. The arrhythmia alarm, important for coronary patients who are susceptible to experiencing arrhythmia, was enabled for a little more than 20% of the patients in the DS and a little more than 46% in the NS. Arrhythmia monitoring is linked to electrocardiographic monitoring and heart rate, however, this equipment depends on various programing steps to reliably detect critical events.

**Table 2 -:** Profiles of alarms that were enabled among the patients under observation and the volume of alarms of the multi-parametric monitors. Rio de Janeiro, RJ, Brazil, 2012

Alarms on	Day Shift (DS) (n=49)	Night Shift (NS) (n=39)
ECG – arrhythmia alarm (%)	10 (20.41%)	18 (46.15%)
Heart rate alarm (%)	45 (91.84%)	39 (100%)
IABP^†^ alarm (%)	23 (46.94%)	10 (25.64%)
NIABP^‡^ alarm (%)	24 (48.98%)	25 (64.10%)
Pulse alarm (%)	1 (2.04%)	0 (0.00%)
Respiratory alarm (%)	18 (36.73%)	4 (3.31%)
SpO_2_^§^ (%)	18 (36.73%)	23 (58.97%)
Volume of the alarms from the multi-parametric monitors -dB (Median and IQR)^||^	75 (60-90)	90 (60-90)

n=Total number of patients monitored under observation in DS (n=49) and in NS (n=39).;

† IABP: Invasive Arterial Blood Pressure;

‡ NIABP: Non- Invasive Arterial Blood Pressure;

§ SpO2: Oxygen saturation.

In regard to the volume of alarms, a median of 75 with an IQR (interquartile range) of 60-90 in the DS and a median of 90 with an IQR of 60-90 was observed in the NS, with no significant variation between the shifts. The volume of the quietest alarm recorded during the DS was 15dB and the loudest was 120dB. During NS, the quietest volume was 45dB and loudest was 120dB.

The profiles of the alarms monitoring the physiological variables and that went off among the patients under observation are presented in Table 3.

**Table 3 -:** Profiles of alarms monitoring physiological variables and which went off. Rio de Janeiro, RJ, Brazil, 2012

Alarms of physiological variables	Day Shift (DS) (n = 106)	Night Shift (NS) (n = 121)
Heart rate alarm (%)	34 (32.08%)	22 (18.18%)
ECG-Arrhythmia alarm^†^ (%)	3 (2.83%)	7 (5.79%)
IABP alarm^‡^ (%)	26 (24.53%)	19 (15.70%)
NIABP alarm^§^ (%)	10 (9.43%)	15 (12.40%)
Respiratory alarm (%)	16 (15.09%)	5 (4.13%)
SpO_2 _alarm^||^ (%)	17 (16.04%)	53 (43.80%)

n= nº total of alarms = 227: DS (n = 106) NS (n = 121);

† ECG-arrhythmia (electrocardiographic tracing);

‡ IABP: Invasive Arterial Blood Pressure;

§ NIABP: Non-Invasive Arterial Blood Pressure;

|| SpO2: Oxygen saturation.

The low percentage of alarms of arrhythmia is because most alarms were not enabled. There was a high percentage of SpO_2 _alarms, especially during the NS.

## Discussion

It is worth noting that a single nurse is not able to meet all requests, demands or system calls^(^ 9 ^)^.

The importance of monitoring critical cardiac patients in order to rapidly visualize clinical changes, identify arrhythmias, bundle branch block, ischemia, and critical heart rates, titration of medications, and control of mechanical ventilator support is unquestionable.

For proper monitoring, however, basic principles should be followed, such as preparing the patient's skin, properly placing electrodes, cables, sensors, and electrical transduction systems, providing proper guidance to the patient, programming and configuring equipment systems, adjusting sensitivity, speed, gain of ECG tracing, derivation that is chosen and indicated according to the patient's cardiac impairment, range of maximum and minimum alarms, detection and rejection of pacemaker pulse, analysis of ST segment and arrhythmia, in addition to filters^(^ 6 ^)^.

The adoption of these principles are recommendations provided by studies and research institutes because they decrease the occurrence of false alarms due to interference; false alarms contribute to desensitization, lack of confidence, and lack of response on the part of the staff, that is, they decrease alarm fatigue^(^ 3 ^,^ 7 ^)^.

Cacophony in the unit, a myriad of alarms from medical devices, creates an environment that poses a significant risk to patient safety. With the accumulation of alarms, it is difficult to identify the origin of a particular alarm, considering the limitations in the ability of human beings to discriminate different categories of sounds in the same environment^ (10)^.

Alarms can go on unendingly and important alarms may be overlooked and intercurrences go unnoticed. Additionally, noise negatively affects the health staff, possibly leading to stress, burnout, conflict, and among patients, noise may cause insomnia, increase duration of hospitalization, and the use of analgesic and anxiolytics^(^ 6 ^)^.

Heeding and resolving the causes of alarms both demand time from the staff, interrupt their tasks and cause distractions that may lead to errors due to a lack of concentration and/or lapses in attention^(^ 11 ^)^. Note that programing, configuring and adjusting alarms is important to meeting the needs of patients. Proper programming ensures that alarms will be valid and warn of truly critical situations, so that the staff can rely on them and decrease unnecessary interruptions and distractions^(^ 12 ^)^.

Alarm overload and "Alarm fatigue" are conditions that may lead to incidents. The staff may deactivate variables that need to be monitored, lower the volume, disable alarms or inadvertently adjust their parameters beyond the limits appropriate for the patients' needs in an attempt to decrease the number of alarms. Such changes may impede the staff from realizing that patients have clinical conditions requiring attention^(^ 7 ^)^.

In regard to volume, the staff should analyze whether the alarms are sufficiently audible in the units, and when programing them, the staff should take into account environmental noise, the number of professionals in the unit, patients, and the unit's physical disposition, in order to adapt the alarms to the needs of each unit^(^ 6 ^)^. Adverse events caused by low-volume alarms have been reported^(^ 2 ^)^.

**Figure 1 -:**
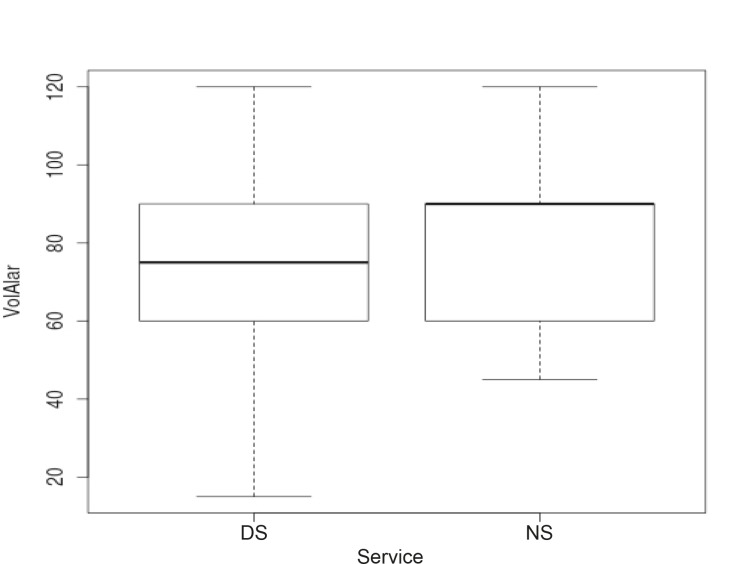
**Boxplot concerning the volume of the alarms of the multi-parametric monitors under observation.** DS - Day Shift NS - Night Shift. Alarm volume: a median 75 with an IQR of 60 - 90 during the DS and a median of 90 with an IQR of 60 - 90 in the NS.

This study's results show that the alarms of monitors under observation were set at a low volume (Figure 1). The monitors' volumes were adjusted from 0 to 255 dB, i.e., the staff can adjust the monitors to a very low volume, which may become inaudible due to the total number of alarms going off within the unit combined with other environmental noise.

The Brazilian Association of Technical Standards (ABNT) establishes levels between 35 and 45 dBA for internal hospital environments (e.g., rooms, nursing wards, nursery, and surgical centers). These norms first take into account auditory comfort and then the acceptable upper and lower limits^(^ 13 ^)^; the same parameters are recommended by the United States Environmental Protection Agency.

The results regarding monitors of physiological variables, the alarms of which were deactivated or the volume was set low, show there is a false sense of security within the unit.

The physiological variables, the alarms of which were enabled, that most frequently went off in both the DS and NS, were heart rate and average Invasive Arterial Blood Pressure (IABP). A high number of alarms monitoring oxygen saturation, especially during the NS, were also observed.

Most alarms observed in a prospective observational study were threshold alarms (70%); i.e., they were out of the pre-set limit and monitored systolic blood pressure (45%), oxygen saturation (19%), heart rate (18%), mean blood pressure (12%), or respiratory frequency (4%). Oxygen saturation generated 90% of the technical alarms^(^ 14 ^)^. Another prospective observational study reports that systolic blood pressure (45.4%) was the variable generating the highest number of alarms followed by oxygen saturation (29.5%)^(^ 14 ^)^.

Alarm fatigue is a challenge because it involves human factors, as well as factors concerning equipment, alarm devices, the internal system of units, and workflow components^(^ 15 ^)^. Its worst consequence is a clinical situation in which there is the real need for immediate care but intervention does not occur because no one pays attention to the alarm, possibly leading patients to experience an adverse event^(^ 16 ^)^

## Conclusion

There is a pressing need to implement safer monitoring in intensive care units to ensure that patients in severe conditions have safe intensive care, otherwise, intensivist professionals, particularly nurses, will be denying Nightingale's teachings upon which intensive care, or more strongly, upon which the intensive care unit itself, is based, the main characteristic of which is monitoring patients. 

Thus, from the perspective of intensive care and based on this study's results, the construct "safe monitoring" emerges. This construct is seen as a way of monitoring, that is, a way of following, tracking, and/or watching the patient in a critical condition through the responsible and rational use of technological resources and alarm systems of medical equipment designed for multi-parametric monitoring and advanced life support, aiming to optimize monitoring and safety in the delivery of intensive care, minimizing risks of an incident that results in harm or an adverse event.

This study's results reinforce our understanding that programming and configuration of physiological variables, volume, and the parameters of alarms of multi-parametric monitors should be incorporated into intensive care units because patients in severe conditions depend on this technological apparatus not only for diagnosis and therapeutic purposes, but also to improve safety. Thus, inappropriate use of this equipment, which may lead to alarm fatigue, may compromise the safety of patients.

It is disturbing that the alarms of equipment intended to protect patients may, in fact, lead to increased noise within the unit and consequently lead to alarm fatigue, distraction and interruption of the workflow and then to a false sense of security.

Through appropriate monitoring, the staff will know the real need to attend to alarms, will trust in the clinical relevance and urgency of these devices, reducing trivialization and over-familiarization with noise. Additionally, patients hospitalized in intensive care units will benefit from measures intended to reduce noise coming from alarms. Therefore, alarms are good for intensive care provided they are properly programed, configured, adjusted, heeded and valued by the staff.
